# Epoetin-β treatment in patients with cancer chemotherapy-induced anaemia: the impact of initial haemoglobin and target haemoglobin levels on survival, tumour progression and thromboembolic events

**DOI:** 10.1038/sj.bjc.6605255

**Published:** 2009-09-29

**Authors:** M Aapro, B Osterwalder, A Scherhag, H U Burger

**Affiliations:** 1 Institut Multidisciplinaire d'Oncologie, Clinique de Genolier, 1, route du Muids, Genolier, Switzerland; 2 F Hoffmann-La Roche Ltd, Basel, Switzerland; 3 Ist Medical Clinic, University Hospital Mannheim, University of Heidelberg, Mannheim, Germany

**Keywords:** anaemia, epoetin-*β*, survival

## Abstract

**Background:**

Epoetin-*β* is used to treat patients with cancer undergoing chemotherapy to alleviate the symptoms of anaemia, reduce the risk of blood transfusions and improve quality of life (QoL).

**Methods:**

This meta-analysis of all 12 randomised, controlled studies of epoetin-*β* evaluated the impact of therapy at different Hb-initiation levels and to different target Hb levels on overall survival, tumour progression and thromboembolic events (TEE). An analysis of risk factors pre-disposing patients to TEEs under epoetin-*β* therapy was also performed. A total of 2297 patients are included in the analysis.

**Results:**

Analyses based on various Hb-initiation levels indicate no detrimental impact on survival (HR 0.99; 95% CI 0.70, 1.40) and a favourable impact on disease progression (HR 0.73; 95% CI 0.57, 0.94) when epoetin-*β* was used within its licensed indication (Hb initiation ⩽10 g dl^−1^) or the EORTC recommended level of 11 g dl^−1^. An increased risk of TEEs is seen for all Hb-initiation level strata and a detrimental impact on survival is seen when initiating epoetin-*β* therapy at Hb levels >11 g dl^−1^. We observe no association between high target Hb levels (⩾13 g dl^−1^) and an increased risk of mortality, disease progression or TEEs with epoetin-*β* compared with control.

**Conclusion:**

The results of this analysis indicate that epoetin-*β* therapy has no detrimental impact on survival or tumour progression when initiated at Hb levels up to 11 g dl^−1^. Furthermore, there is no evidence to suggest that high Hb values achieved during epoetin-*β* therapy are associated with an increased mortality, disease progression or TEE rate.

Anaemia frequently occurs in patients with cancer either as a result of the underlying malignancy or as a consequence of myelosuppressive chemotherapy or radiotherapy, or due to a combination of both (Bokemeyer *et al*, 2005). The symptoms of anaemia have a significant impact on a patient's condition and QoL (Ludwig *et al*, 2004) and as an independent prognostic factor, anaemia is associated with adverse outcomes in patients with a variety of malignancies (Caro *et al*, 2001).

Erythropoiesis-stimulating agents (ESAs) increase Hb levels and reduce transfusion requirements in patients with cancer (Littlewood *et al*, 2001; Österborg *et al*, 2002; Vansteenkiste *et al*, 2002). In addition, improvement in patients' QoL when compared with placebo or standard transfusion therapy was also shown following ESA therapy (Littlewood *et al*, 2001; Crawford *et al*, 2002; Boogaerts *et al*, 2003).

Pre-clinical data have suggested an enhanced tumour response and delayed tumour progression associated with ESA treatment (Mittelman *et al*, 2001; Thews *et al*, 2001; Stuben *et al*, 2003). Moreover, in clinical studies, a potential survival benefit has been shown in patients undergoing cancer therapy who received treatment with ESAs (Antonadou *et al*, 2001; Glaser *et al*, 2001; Littlewood *et al*, 2001). The first of two meta-analyses of controlled trials in cancer patients receiving ESAs, reported by the Cochrane Group, showed a trend toward increased survival in patients treated with ESAs and supported these findings (Bohlius *et al*, 2005). In the second meta-analysis (57 trials including 9353 patients), however, a shift of the hazard ratio for survival towards an increased risk for patients receiving various ESAs was shown (Bohlius *et al*, 2006, 2008).

An association between erythropoietin treatment and increased mortality was originally suggested by two studies in cancer patients (Henke *et al*, 2003; Leyland-Jones *et al*, 2005) raising concerns about the safety of ESAs when targeting high Hb levels (12–14 g dl^−1^ or higher) (Luksenburg *et al*, 2004). In addition, three studies reporting a detrimental impact of ESA treatment on survival have recently been published (Overgaard *et al*, 2007; Wright *et al*, 2007; Smith *et al*, 2008), although one of these studies (Overgaard *et al*, 2007) remains to be reported in full. Methodological limitations of the clinical studies aimed at maintaining a high Hb level in cancer patients (Henke *et al*, 2003; Leyland-Jones *et al*, 2005), may have confounded the results and influenced the findings (Leyland-Jones and Mahmud, 2004; Vaupel and Mayer, 2004). Studies evaluating different haematocrit levels in end-stage renal failure patients with cardiovascular risk factors (Besarab *et al*, 1998; Luksenburg *et al*, 2004) have suggested that increased mortality may be because of a higher risk of thromboembolic events (TEEs) under ESA therapy. Hypotheses that ESAs may promote tumour growth through erythropoietin receptor activation, stimulation of angiogenesis or through hypoxia improvement through Hb increases have also been proposed (Kelleher *et al*, 1998; Acs *et al*, 2001, 2002; Arcasoy *et al*, 2002; Yasuda *et al*, 2003; Janecka, 2004; Vaupel and Mayer, 2004).

We previously reported results of an updated meta-analysis of 12 randomised, controlled studies of epoetin-*β* conducted in 2301 patients undergoing cancer therapy (Aapro *et al*, 2008a) including three recently completed trials with longer term follow-up in patients with head and neck cancer (Henke *et al*, 2003), patients with metastatic breast cancer (Aapro *et al*, 2008b) and patients with cervical cancer (Strauss *et al*, 2008). The results of this meta-analysis based on individual patient level data showed no statistically significant difference between patients receiving epoetin-*β* or control (standard treatment) in terms of overall survival, a favourable trend with respect to the risk of disease progression for patients receiving epoetin-*β* and a higher risk of thromboembolic events associated with epoetin-*β* treatment (Aapro *et al*, 2008a). Recently, the Cochrane Collaboration has published an updated meta-analysis, which includes data on epoetin-*α*, epoetin-*β* and darbepoetin from 53 randomized, controlled studies in cancer patients. The data from this updated meta analysis, suggested a negative effect on overall survival in the overall study population, however, in those patients receiving cancer chemotherapy, no significant adverse effects on overall survival were observed (Bohlius *et al*, 2009).

As a consequence of the safety concerns raised by some studies, the European Health Authorities requested the product labels for marketed ESAs to be restricted to a Hb-initiation level <10 g dl^−1^ and a Hb target not to exceed 12 g dl^−1^. Furthermore, the European Health Authorities have stated that transfusions should be seen as the preferred option. The updated EORTC treatment guidelines, however, recommend the initiation of ESA therapy at Hb levels of between 9 and 11 g dl^−1^ and a sustained Hb level of ∼12 g dl^−1^ should be the target for treatment with ESAs (Bokemeyer *et al*, 2007; Aapro and Link, 2008). Below a Hb level of 9 g dl^−1^, blood transfusions followed by ESA treatment should be considered and prophylactic use of erythropoietins in subjects with normal Hb levels scheduled to undergo chemotherapy or radiotherapy is not recommended.

The objectives of this analysis are to evaluate the impact on overall survival, disease progression and TEEs of different Hb intervention and target levels for epoetin-*β* therapy and in particular, to explore the safety of epoetin-*β* with respect to its effects on overall survival and disease progressions when used within the Hb intervention and target levels as recommended in the revised European label. These data have not been reported as yet and are considered of considerable relevance for prescribing physicians. Furthermore, the influence of baseline prognostic factors on the observed epoetin-*β* effect with respect to time to thromboembolic event in the pooled patient population (*N*=2301) from 12 controlled epoetin-*β* trials in cancer patients was assessed.

## Materials and methods

Data presented in this article are derived from an updated meta-analysis of 12 controlled studies designed to evaluate differences between epoetin-*β* and control (placebo or standard care) with regard to overall survival, disease progression and TEEs during and up to 28 days after end of therapy with epoetin-*β*.

Eligible studies included all randomised, controlled studies of epoetin-*β* conducted by the drug sponsor (F Hoffmann-La Roche (Basel, Switzerland) or Boehringer Mannheim) in patients with cancer undergoing treatment (chemotherapy (seven studies), surgery (two studies), radiotherapy (two studies) or radio-chemotherapy (1 study)). The meta-analysis is based on data derived at the individual patient level. Individual study details are summarised in Table 1.

As most of the studies were originally designed to evaluate the efficacy of epoetin-*β* with respect to anaemia correction, in the majority there was no follow-up for survival or tumour progression beyond study treatment plus a standard 28-day period used to assess SAEs, deaths and disease progression. Furthermore, tumour status was not prospectively assessed in many of the earlier trials with short-term follow-up and details of disease progression were routinely reported as adverse events. For the meta-analysis, this information was analysed retrospectively by reviewers blinded to treatment assignment. Four of the studies, which did evaluate the effects of epoetin-*β* on survival and/or disease progression (Henke *et al*, 2003; Österborg *et al*, 2005; Aapro *et al*, 2008b) or response to treatment (Strauss *et al*, 2008) provided long-term (up to 60 months) follow-up information for overall survival or tumour progression. For the assessment of TEEs, all reported adverse events were reviewed against a pre-specified list of TEEs, which was applied consistently across all studies.

### Statistical analyses

Overall survival, time to progression and time to TEE were analysed by Kaplan–Meier estimates in which the data were stratified; (1) by Hb intervention level (baseline Hb level), and (2) by maximum Hb level achieved up to 28 days after end of treatment (target Hb level).

Two sets of analyses were performed. One set of analyses included individual patient data from all 12 studies. For these analyses, patients without events were censored at 4 weeks after the last entry in the administration record. A second set of analyses using only pooled patient data from the studies with long-term follow up in which all events were included in the analysis was performed for overall survival (Henke *et al*, 2003; Österborg *et al*, 2005; Strauss *et al*, 2008; Aapro *et al*, 2008b) and time to progression (Henke *et al*, 2003; Strauss *et al*, 2008; Aapro *et al*, 2008b). In the study by Österborg *et al*, 2005, patients were followed for survival but not for disease progression, therefore this study was excluded from the time to progression analyses. Patients without an event were censored at the time of last follow-up or, if no follow-up information was available, 4 weeks after the last entry in the administration record. Overall analyses were performed, unstratified as well as stratified by the study.

Risk factors for TEEs and their influence on the observed epoetin treatment effect were also investigated. Potential risk factors were defined at baseline and, as a first step, univariate models estimated the effect of the prognostic factor as well as a corrected treatment effect. Factors that were statistically significant in the univariate model or showed a trend at an *α*-level of 15% were then selected and patients classified into risk subgroups based on clinically established risk profiles. The impact of these selected factors on time to TEE was then analyzed in Cox regression models adjusting by subgroup for each factor separately, for subgroups of patients with no risk factors, one, two and three or more risk factors at baseline to investigate whether a potential risk for TEE in combination with epoetin-*β* treatment was attributable to a particular subgroup of patients at specific risk.

## Results

### Analysis populations

The 12 randomized, controlled trials enrolled a total of 2301 patients of whom 2297 (epoetin-*β*, *n*=1244; control, *n*=1053) were included in the analysis; four patients were excluded because of not receiving treatment of any kind during the trials. In the studies, three patients in the epoetin group received no epoetin-*β* and five patients randomised to control received epoetin-*β*. Patients were analysed according to the treatment received.

### Baseline characteristics and follow-up

Baseline characteristics of the patients in the analysis are described in the earlier publication (Aapro *et al*, 2008a) and are shown in Table 2. Of the 2297 patients in the analysis, 35% had non-myeloid haematological malignancies (mainly non-Hodgkin's lymphoma or multiple myeloma) and 65% had solid tumours (most commonly primary malignancies of the breast, head and neck, colon/rectum and ovary). Other than the slightly higher proportion of patients in the epoetin group with ovarian carcinoma as a result of the three arm design of the study by ten Bokkel Huinink *et al* (1998), no clinically relevant differences between the groups were noted.

Median initial weekly epoetin-*β* dose was 27 000 IU (range 0–90 000 IU). Mean baseline Hb level was 10.6 g dl^−1^ in the control arm and 10.5 g dl^−1^ in the epoetin-*β* arm. During treatment, mean maximum Hb level was 13.4 g dl^−1^ in the epoetin-*β* arm and 12.0 g dl^−1^ in the control arm. The mean baseline adjusted Hb area under the curve was 1.24 g dl^−1^ with epoetin-*β* compared with 0.07 g dl^−1^ with control.

Duration of follow up across the 12 studies was comparable in the epoetin-*β* (median 3.9 months) and control (median 3.8 months) treatment groups (patients without events from the four studies with long-term follow up were censored 4 weeks after last entry in the administration record). In the four studies with long-term follow-up data, when all events were included, median follow up was also comparable (28.8 months with epoetin-*β* and 29.8 months with control) (Aapro *et al*, 2008a).

### Impact of baseline Hb level on overall survival

As previously described in Aapro *et al* (2008a), there was no statistically significant difference between patients receiving epoetin-*β* or control (standard treatment) in terms of overall survival in the unstratified pooled analysis of all 12 controlled studies (HR of 1.13, 95% CI 0.87; 1.46, log-rank *P*-value=0.355, *N*=2297). Comparable results were found in the unstratified pooled analysis of the four studies with long-term follow up (HR of 1.13 (95% CI 0.98; 1.31, log-rank *P*-value=0.082, *N*=1227).

**Figure 1:**
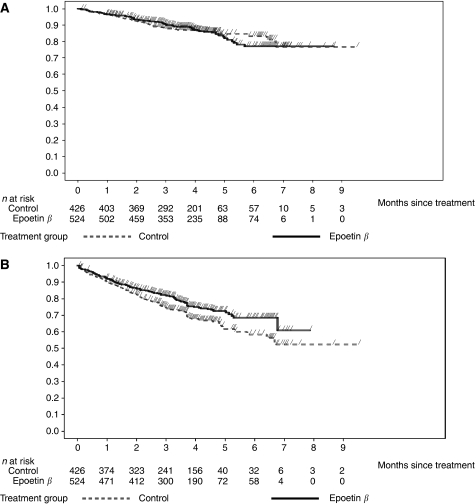
Kaplan–Meier time to event analyses of (**A**) overall survival and (**B**) time to progression in patients with Hb-initiation levels ⩽10 g dl^−1^.

**Figure 2:**
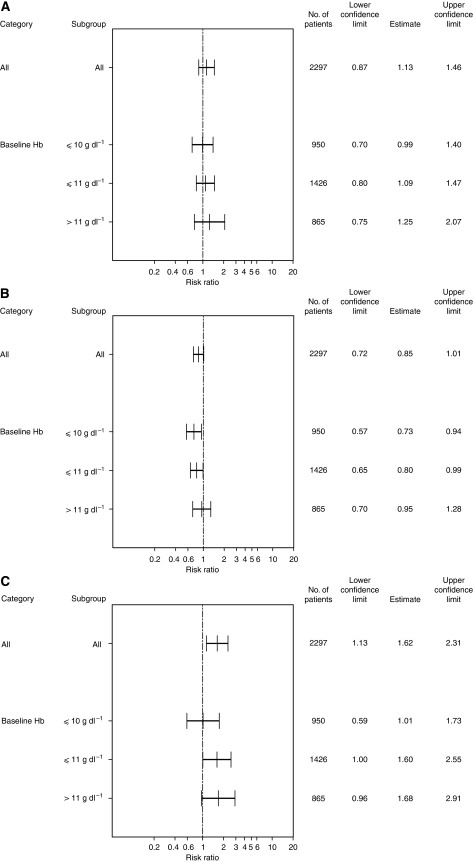
Subgroup analysis of hazard ratios for (**A**) overall survival, (**B**) time to progression and (**C**) time to TEE by Hb-initiation level.

When stratified by baseline Hb level, the time to event analyses in patients with baseline Hb levels ⩽10 g dl^−1^ showed a HR of 0.99 (95% CI 0.70; 1.40; log-rank *P*-value=0.96; *N*=950) (Figures 1A and 2A) and for patients with baseline Hb levels ⩽11 g dl^−1^ a HR of 1.09 (95% CI 0.80; 1.47; *N*=1426) (Figure 2A). These results are within the range of those for the unstratified pooled population. However, for patients whose baseline Hb levels were above 11 g dl^−1^, the mortality risk appeared to be higher (HR of 1.25, 95% CI 0.75; 2.07; *N*=865) (Figure 2A).

Comparable results were found in the pooled analysis of four studies with long-term follow up (Table 3). These data suggest no increased risk of epoetin-*β* treatment on overall survival in patients whose baseline haemoglobin was ⩽10 g dl^−1^ (HR=0.91, 95% CI 0.72; 1.16) or ⩽11 g dl^−1^ (HR=1.03, 95% CI 0.85; 1.25), which was consistent with the unstratified analysis (HR=1.13, 95% CI 0.98; 1.31, log-rank *P*-value=0.082). In patients whose baseline haemoglobin levels were above 11 g dl^−1^, however, the risk of mortality appeared to increase (HR=1.24, 95% CI 1.00; 1.53).

### Impact of baseline Hb levels on disease progression

In the unstratified pooled analysis of 12 controlled studies, no significant differences between the epoetin-*β* and control groups were seen in the number of patients with disease progression (Aapro *et al*, 2008a). Kaplan–Meier analysis indicated a reduced risk of progression for patients treated with epoetin *β* compared with control (HR=0.85, 95% CI 0.72; 1.01, log-rank *P*-value=0.072).

When stratified by baseline Hb level, the subgroup with a baseline Hb level ⩽10 g dl^−1^ had a relative risk reduction for disease progression of 27% (HR 0.73, 95% CI 0.57; 0.94; log-rank *P*-value=0.013; *N*=950) (Figures 1B and 2B) and the subgroup with a baseline Hb level ⩽11 g dl^−1^ had a relative risk reduction for disease progression of 20.0% (HR 0.80, 95% CI 0.65; 0.99; *N*=1426) with epoetin-*β* compared with control (Figure 2B). These results are consistent with the results for the pooled population (relative risk reduction of 15%). For patients with baseline Hb values >11 g dl^−1^, there was no evidence of an increased risk of disease progression associated with epoetin-*β* treatment (HR 0.95, 95% CI 0.70; 1.28) (Figure 2B).

In the three studies with long-term follow up of disease progression, results suggested favourable effects of epoetin-*β* over control with respect to disease progression in the subgroups with a baseline Hb level ⩽10 g dl^−1^ (HR=0.59, 95% CI 0.36; 0.96) and a baseline Hb level ⩽ 11 g dl^−1^ (HR=0.85, 95% CI 0.64; 1.13; Table 3). This finding contrasts with a 13% higher risk of disease progression for the unstratified pooled population. For subgroups with baseline Hb levels >11 dl^−1^, the relative risk for disease progression with epoetin-*β* increased by 30% compared with control (Table 3).

### Impact of baseline Hb levels on time to TEE

Across the 12 studies in the unstratified pooled analysis, a higher TEE event rate was observed in the epoetin-*β* group compared with the control (0.22 *vs* 0.14 events/patient year) with an overall HR for time to TEE of 1.62 (95% CI 1.13; 2.31, log-rank *P*-value=0.008) (Aapro *et al*, 2008a).

When stratified by baseline Hb level (Figure 2C) an increased risk of thromboembolic events with epoetin-*β* compared with control was seen across all baseline Hb strata subgroups; the risk being lowest in patients with baseline Hb levels ⩽10 g dl^−1^ and increasing with the higher the baseline Hb level.

### Impact of maximum-achieved Hb levels on survival, disease progression and TEEs

**Figure 3:**
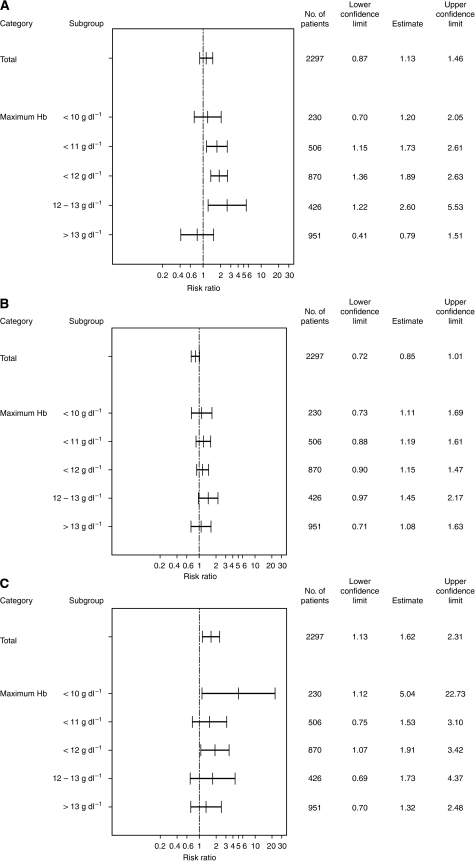
Subgroup analysis of hazard ratios for (**A**) overall survival, (**B**) time to progression and (**C**) time to TEE by maximum-achieved Hb level.

The hazard ratios for overall survival, time to progression and time to TEE stratified by maximum-achieved Hb level for the pooled population of 12 studies are shown in Figure 3. These data indicate a shift towards an increased mortality risk for subgroups with maximum-achieved Hb values between 10 and <13 g dl^−1^ (HR range: 1.20–2.60 for overall survival (Figure 3A); 1.11–1.45 for disease progression (Figure 3B); 1.53–5.04 for time to TEE (Figure 3C)). However, there was no indication for an increased risk of mortality, disease progression or TEEs in patients achieving maximum Hb levels >13 g dl^−1^ (HR=0.79, 1.08 and 1.32, respectively) when compared with the unstratified analyses (Figure 3).

In the pooled analyses of studies with long-term follow up (Table 3), a shift of the hazard ratio towards an increased mortality risk was seen for subgroups with maximum-achieved Hb values between 10 and <12 g dl^−1^ (HR range 1.71–1.89 for overall survival). However, for subgroups with maximum-achieved Hb levels ⩾12 g dl^−1^ (HR range 1.50–1.59) there was no indication for a further increased risk of mortality.

With regards to disease progression in the three studies with long-term follow up, a shift in the hazard ratios towards an increased risk of progression was seen in all subgroups with maximum-achieved Hb values of between 10 and ⩽13 g dl^−1^. However, there was no indication of an increased risk in the subgroup of patients achieving maximum Hb levels >13 g dl^−1^ (Table 3).

### Risk factors for thromboembolic events

Risk factors and their influence on the epoetin-*β* treatment effect with respect to TEEs were investigated in a Cox regression model into which treatment and other prognostic factors were added (Table 4). Five factors; previous history of TEEs, previous coronary artery disease, baseline hypertension, baseline dyslipidemia and age at baseline >65 years were statistically significant or showed a trend at an *α*-level of 15% in these univariate models. Adjusted treatment effects remained unchanged. Results of the Cox regression analysis based on the number of risk factors at baseline indicated that the risk of TEEs was generally higher in patients with two or more risk factors compared to those with fewer than two risk factors; Table 4). Overall analyses stratified by study yielded similar results.

## Discussion

### Impact of baseline Hb level

We reported previously that in an unstratified overall analysis, patients in the epoetin-*β* group had a numerically, but statistically non-significant increased risk of mortality compared with patients in the control group (Aapro *et al*, 2008a). These results are consistent both with those of a recently published, individual patient data-based meta-analysis by the Cochrane Collaboration (Bohlius *et al*, 2009), which showed no negative effect of epoetin therapy in patients receiving chemotherapy, and with those from a subset of patients receiving chemotherapy in a meta-analysis published by Tonelli *et al* (2009). In contrast to our results, however, in the overall study population of the meta-analysis published by the Cochrane Collaboration, which included patients not receiving chemotherapy, a detrimental effect on overall survival was observed.

When stratified by baseline Hb level, no significantly increased negative effects on mortality were seen for the epoetin-*β* group up to a baseline Hb level of 11 g dl^−1^. Above this baseline Hb level, the risk of mortality increased for epoetin-*β* patients compared with control patients. The robustness of these findings was confirmed in the pooled analyses of four studies, which collected long-term follow-up survival data (1227 patients) in which an increased risk of mortality was only seen in the subgroup of patients initiating epoetin-*β* therapy with a baseline Hb >11 g dl^−1^.

These results support the current labelling for epoetin-*β*, which recommends a Hb-initiation level of ⩽10 g dl^−1^ and the updated EORTC treatment guidelines (Bokemeyer *et al*, 2007; Aapro and Link, 2008), which recommend a Hb-initiation level of ⩽ 11 g dl^−1^.

Comparable results have been reported by the Cochrane Group from an aggregated study data-based meta-analysis of ESA studies (Bohlius *et al*, 2006), where no significantly increased risk of mortality was seen between the ESA and control groups in those studies, which enrolled patients with baseline Hb levels <10 g dl^−1^ or in studies enrolling patients with baseline Hb levels 10–12 g dl^−1^. However, in studies enrolling patients with baseline Hb levels above this level (7 trials; 1696 patients) an increased risk of death was seen in the ESA group.

A favourable outcome with respect to disease progression with ESAs *vs* control was previously shown by the Cochrane Collaboration (Bohlius *et al*, 2006), and in a systematic review of 46 ESA trials conducted for the National Institute for Clinical Excellence (NICE) (Wilson *et al*, 2007). Consistent with these findings, in this analysis, epoetin-*β* treatment showed a reduced risk of disease progression both in the overall unstratified analysis and when restricted to three studies with long-term follow up for disease progression (*N*=884). Analyses of the overall pooled population stratified by baseline Hb level showed similar favourable effects of epoetin-*β vs* control on disease progression in subgroups with a baseline Hb level of ⩽10 g dl^−1^, thus supporting the current label, and subgroups with a baseline Hb level ⩽11 g dl^−1^, consistent with current EORTC guidelines (Aapro and Link, 2008). In the stratified pooled analyses of the long-term follow-up studies, the favourable effect on disease progression was maintained for all subgroups, including those with baseline Hb levels >11 g dl^−1^. These results suggest, therefore, that when epoetin-*β* therapy is used according to its current label and following the updated EORTC guidelines there is no increased risk of disease progression *vs* control.

### Impact of maximum-achieved Hb level

The increased mortality associated with erythropoietin treatment in cancer patients reported by Henke *et al* (2003); Leyland-Jones *et al* (2005), and more recently in studies by Overgaard *et al* (2007); Wright *et al* (2007); Smith *et al* (2008), is not supported by the present meta-analysis. Subgroup analyses based on maximum-achieved Hb level during epoetin-*β* treatment do not provide evidence for an increased risk of mortality, disease progression or TEEs with epoetin-*β* compared with control in patients achieving Hb levels >13 g dl^−1^, although we observe a shift in the hazard ratios towards an increased risk for all three events associated with maximum-achieved Hb values between 10 and 13 g dl^−1^.

The results of these analyses need to be interpreted with caution because of the associated methodological limitations and potential confounding influence of factors such as baseline disease status, that are related to outcome and have a strong influence on the likelihood of achieving a better response to treatment in terms of Hb increase. Furthermore, the comparisons undertaken here were not based on randomized groups and hence comparisons in each of the subgroups are limited by the lack of a respective control. For example, for the subgroup of patients with maximum-achieved Hb levels <10 g dl^−1^, comparisons are made between patients receiving epoetin-*β* who, despite treatment, did not respond to therapy and patients in the control group who remained below 10 g dl^−1^ without treatment. In contrast, in the subgroup of patients with maximum-achieved Hb levels >13 g dl^−1^, the comparison is between those patients who were titrated to beyond 13 g dl^−1^ with epoetin-*β* therapy and those who either achieved Hb levels above 13 g dl^−1^ without treatment or who were enrolled with baseline Hb levels above 13 g dl^−1^. On the assumption that an increase in Hb levels independent of epoetin-*β* treatment is a good prognostic factor, these analyses would therefore be biased in favour of the control group. Nonetheless, no convincing evidence was found in the present analyses to suggest that high Hb values achieved with epoetin-*β* therapy are associated with an increased risk of death, disease progression or TEEs.

### Risk factors for thromboembolic events

The present analyses show a significantly increased TEE rate with epoetin-*β* compared with control (0.22 events/patient year *vs* 0.14 events/patient year) and an increased risk of thromboembolic events with epoetin-*β*. These results are consistent with those reported in both meta-analyses of the Cochrane Collaboration (Bohlius *et al*, 2005, 2006). Subgroup analyses based on Hb-initiation level indicate a correlation between Hb-initiation level and risk of TEE. This increased TEE risk is well documented within the ESA class in general and adequately addressed in the product labelling for all approved ESAs.

Among the multiple risk factors shown for thromboembolic disease, the most influential include increasing age, prolonged immobility, malignant disease, major surgery, multiple trauma, previous venous thromboembolism and chronic heart failure (Anderson and Spencer, 2003). Increased TEE risk has been suggested to be related not only to the presence of these individual risk factors, but also to the number of pre-disposing risk factors at baseline (Nicolaides and Irving 1975; Wheeler *et al*, 1982). In the present analysis five major pre-disposing risk factors for TEEs in patients receiving epoetin-*β* therapy were identified (age >65 years, previous thromboembolic event, coronary artery disease, hypertension and dyslipidemia). Furthermore, the combination of two or more of these risk factors in epoetin-*β*-treated patients markedly increased the risk of a TEE. Recently, an association between RBC and platelet transfusions and an increased risk of TEEs and mortality in cancer patients has also been suggested (Khorana *et al*, 2008). Further investigation of this potential causal relationship in patients included in the epoetin-*β* studies is merited.

The results could suggest a potential role for the use of prophylactic antithrombotic agents in patients with known risk factors who are scheduled to receive epoetin-*β* therapy (Aapro *et al*, 2009). Further investigation is required to confirm the benefits of such treatment in combination with epoetin-*β*. These results concerning the increased risk for TEEs with epoetin-*β* are consistent with the observation and conclusions of the Cochrane meta-analyses (Bohlius *et al*, 2005, 2006) and are currently addressed in the labelling recommendations for epoetin-*β*.

## Conclusions

This individual patient data-based meta-analysis of 12 controlled epoetin-*β* studies in cancer patients is the first to enable subgroup analyses assessing the risk of ESA therapy associated with different Hb-initiation levels. Results indicate that when used within the terms of its licensed indication (i.e., Hb-initiation level ⩽10 g dl^−1^) or within the updated EORTC guidelines (i.e., Hb-initiation level ⩽11 g dl^−1^), there was no evidence of an increased risk of mortality or disease progression associated with epoetin-*β* therapy. Consistent with other published meta-analyses, the thromboembolic event rate was significantly increased. The number and combination of pre-disposing risk factors for TEEs at baseline should be taken into account before initiating epoetin-*β* therapy, and potential preventative measures should be considered. Although there is a clear trend towards a higher risk of overall mortality, disease progression and thromboembolic event rate was observed with increasing baseline Hb values, such a trend was not seen in patients who achieved higher Hb values, especially when Hb values above 13 g dl^−1^ were exceeded during therapy.

## Figures and Tables

**Table 1:** Main features of randomised clinical trials of epoetin-*β* in patients with cancer

**Study**	**Design and no. of patients (epoetin**-*β***/ control)**	**Diagnosis**	**Epoetin**-***β*** **dosage and duration of therapy**	**Control**	**Cancer treatment**
ten Bokkel Huinink *et al* (1998)	o, pg *n*=*83/87*	Ovarian cancer, Hb <13 g dl^−1^	150 or 300 IU kg^−1^ 3 × week × 6 months	Standard therapy	Chemotherapy
Österborg *et al* (1996)	o, pg *n*=*95/49*	MM, NHL, CLL; transfusion-dependent, Hb <10 g dl^−1^	2000–10 000 IU day^−1^ titrated or 10 000 IU day^−1^ fixed dosage × 24 weeks	Standard therapy	Chemotherapy
Rau *et al* (1998)	db, pc and pg *n*=*28/26*	Resectable rectal cancer, Hb ⩾ 12.5 g dl^−1^ (men), ⩾12 g dl^−1^ (women)	200 IU kg^−1^ daily × 11 days	Placebo	Surgery
Kettelhack *et al* (1998)	db, pc *n*=*52/57*	Colorectal cancer suitable for hemicolectomy, Hb >8.5–13.5 g dl^−1^	20 000 IU day^−1^ × 10–15 days	Placebo	Surgery
Data on file (study MF4266)	o, pg *n*=*10/10*	AML	10 000 IU day^−1^, then weekly or twice weekly × ⩽30 weeks	Standard therapy	Chemotherapy
Cazzola *et al* (1995)	o, pg *n*=*117/29*	MM, NHL, CLL; transfusion-independent, Hb⩽11 g dl^−1^	1000, 2000, 5000 or 10 000 IU day^−1^ × 8 weeks	Standard therapy	Chemotherapy
Oberhoff *et al* (1998)	pg, *n*=*114/104*	Solid organ tumours, Hb⩽11 g dl^−1^	5000 IU day^−1^ × 12–24 weeks	Standard therapy	Chemotherapy
Boogaerts *et al* (2003	o, pg *n*=*131/128*	Malignant disease, Hb⩽11 g dl^−1^	150 IU kg^−1^ 3 × week adjusted for Hb response × 12 weeks	Standard therapy	Chemotherapy
Österborg *et al* (2002, 2005)	pc, db and pg *n*=*170/173*	MM, NHL, CLL; transfusion-dependent and epo-deficient, Hb⩽10 g dl^−1^	150 IU kg^−1^ 3 × week adjusted for Hb response × 16 weeks 12-month study period^a^	Placebo	Chemotherapy
Henke *et al* (2003)	pc, db and pg *n*=*171/180*	Head and neck cancer, Hb<13 g dl^−1^ (men), <12 g dl^−1^ (women)	300 IU kg^−1^ 3 × week, 6–8 weeks 60-month study period	Placebo	Radiotherapy
Strauss *et al* (2008)	o, pg *n*=*34/40*	Cervical cancer Stage FIGO IIB-IVA, Hb 9–13 g dl^−1^	150 IU kg^−1^ 3 × week, 8–14 weeks, 6-month study period	Standard therapy	Radio-chemotherapy
Aapro *et al* (2008b)	o, pg *n*=*231/232*	Breast cancer, Hb<12.9 g dl^−1^	30 000 IU weekly × 24 weeks, 24-month study period	Standard therapy	Chemotherapy

Abbreviations: AML=acute myeloid leukaemia; CLL=chronic lymphocytic leukaemia; db=double-blind; Hb=haemoglobin; MM=multiple myeloma; NHL=non-Hodgkin's lymphoma; o=open design; pc=placebo controlled; pg=parallel group. Patients had anaemia unless stated otherwise, and standard therapy consisted of antitumour treatment plus blood transfusion as required.

Reproduced from Aapro *et al*, 2008a.

a Information on disease progression not collected during the follow-up period of this study.

**Table 2:** Baseline characteristics of pooled study populations

**Parameter**	**Control (*n*=**1053)**	**Epoetin-*β* (*n*=**1244)**
*Gender (Percentage of male/female)*	37/63	38/62
		
*Race*		
*n*	921	1069
Caucasian	882 (96%)	1029 (96%)
Other	39 (4%)	40 (4%)
		
*Mean age in years (range)*	58.8 (19–91)	59.3 (20–87)
*Mean weight in kg (range)*	67.7 (30.0–131.5)	67.1 (35.0–118.0)
*n*	1048	1235
		
*Mean height in cm (range)*	166.7 (140–198)	166.4 (126–198)
*n*	809	1012
		
*Tumour type,* n (%)		
Haematological	331 (31.4)	465 (37.4)
Acute myeloid leukaemia	10 (3.0)	10 (2.2)
Multiple myeloma	125 (37.8)	204 (43.9)
Non-Hodgkin's lymphoma	195 (58.9)	247 (53.1)
Hodgkin's lymphoma	1 (<1)	4 (<1)
		
*Solid*	722 (68.6)	779 (62.6)
Breast	261 (36.2)	261 (33.5)
Head/neck	174 (24.1)	181 (23.2)
Gynaecological	133 (18.4)	186 (23.9)
Gastrointestinal	96 (13.3)	100 (12.8)
Other	58 (8.0)	51 (6.6)
		
*Haemoglobin*		
*n*	1050	1241
Mean (range)	10.6 (5.7–16.7)	10.5 (4.2–17.1)
Median	10.5	10.4

Data were collected from all 2297 patients unless otherwise stated.

Reproduced from Aapro *et al*, 2008a.

**Table 3:** Hazard ratios for overall survival, time to progression and TEE by Hb-initiation level or maximum-achieved Hb level in long-term follow-up studies

	**Overall survival**			**Time to progression**		
**Patient subgroup Hb-initiation level**	**Number of patients**	**Hazard ratio (95% CI)**	***P*-value^a^**	**Number of patients**	**Hazard ratio (95% CI)**	***P*-value^a^**
*Long-term follow-up studies*						
All patients	1227	1.13 (0.98; 1.31)	0.082	884	1.13 (0.95; 1.34)	0.165
⩽10 g dl^−1^	393	0.91 (0.72; 1.16)		114	0.59 (0.36; 0.96)	
⩽11 g dl^−1^	612	1.03 (0.85; 1.25)		285	0.85 (0.64; 1.13)	
>11 g dl^−1^	611	1.24 (1.00; 1.53)		595	1.30 (1.05; 1.61)	
						
*Maximum Hb value*						
*Long-term follow-up studies*						
All patients	1227	1.13 (0.98; 1.31)	0.082	884	1.13 (0.95; 1.34)	0.165
<10 g dl^−1^	64	1.71 (0.97; 3.02)		16	10.17 (0.99; 104.36)	
10–<11 g dl^−1^	163	2.09 (1.42; 3.08)		60	2.37 (1.01; 5.57)	
11–<12 g dl^−1^	352	1.89 (1.45; 2.46)		166	1.85 (1.14; 2.98)	
12–13 g dl^−1^	259	1.59 (1.15; 2.23)		191	1.75 (1.13; 2.69)	
>13 g dl^−1^	599	1.50 (1.16; 1.94)		511	1.83 (1.39; 2.41)	

^a^HR *P*-value (Wald test).

**Table 4:** Risk analysis for time to thromboembolic event

		**Covariate factor effect**		**Treatment effect adjusted by covariate**	
**Covariate**	***n***	**Hazard ratio (95% CI)**	***P*-value^a^**	**Hazard ratio (95% CI)**	***P*-value^a^**
Treatment effect (unadjusted)	2297	—	—	1.62 (1.13; 2.31)	0.0081
Gender	2297	1.09 (0.76; 1.57)	0.637	1.62 (1.14; 2.32)	0.0078
Previous thromboembolic event	2297	2.23 (1.13; 4.38)	0.021^b^	1.64 (1.15; 2.35)	0.0065
Coronary artery disease	2297	1.99 (0.88; 4.52)	0.098^b^	1.62 (1.14; 2.32)	0.0079
Hypertension	2297	1.57 (1.08; 2.27)	0.018^b^	1.62 (1.13; 2.31)	0.0082
Diabetes	2297	1.16 (0.62; 2.14)	0.643	1.62 (1.13; 2.32)	0.0080
Dyslipidemia	2297	1.96 (0.86; 4.44)	0.108^b^	1.64 (1.14; 2.34)	0.0069
Previous MI, angina, unstable angina	2297	1.16 (0.54; 2.48)	0.704	1.62 (1.13; 2.31)	0.0081
Obesity	2297	0.76 (0.46; 1.25)	0.286	1.62 (1.14; 2.32)	0.0077
Age >65 years at baseline	2297	1.35 (0.96; 1.91)	0.085^b^	1.62 (1.13; 2.31)	0.0084

	**Control**			**Epoetin-*β***			
**Subgroup**	***n***	**Number of events**	**KM 90 day event rate**	***n***	**Number of events**	**KM 90 day event rate**	**Hazard ratio (95% CI)**
*All patients*	1053	46	0.96	1244	88	0.94	1.62 (1.13; 2.31)
No risk factors	555	20	0.96	646	36	0.96	1.54 (0.89; 2.66)
One risk factor	348	22	0.93	431	31	0.93	1.09 (0.63; 1.88)
Two risk factors	125	2	0.99	136	13	0.90	6.35 (1.43; 28.13)
Three or more risk factors	25	2	0.92	31	8	0.77	3.33 (0.71; 15.69)

a HR *P*-value (Wald test).

b Covariates significant at the 15% level in univariate analysis.
